# The ontogeny of choanocyte chambers during metamorphosis in the demosponge *Amphimedon queenslandica*

**DOI:** 10.1186/s13227-016-0042-x

**Published:** 2016-03-08

**Authors:** Shunsuke Sogabe, Nagayasu Nakanishi, Bernard M. Degnan

**Affiliations:** School of Biological Sciences, University of Queensland, Brisbane, QLD 4072 Australia

**Keywords:** Porifera, Choanocyte, Choanocyte chamber, Development, Demospongiae, Archeocyte, Invertebrates, Evolution, Stem cells, Ontogeny

## Abstract

**Background:**

The aquiferous body plan of poriferans revolves around internal chambers comprised of choanocytes, a cell type structurally similar to choanoflagellates. These choanocyte chambers perform a range of physiological and developmental functions, including the capture of food and the generation of stem cells. Despite the increasing interest for choanocytes as sponge stem cells, there is limited knowledge on the development of choanocyte chambers. Using a combination of cell lineage tracing, antibody staining and EdU labeling, here we examine the development of choanocytes and the chambers they comprise during metamorphosis in the marine demosponge *Amphimedon queenslandica*.

**Results:**

Lineage-tracing experiments show that larval epithelial cells transform into mesenchymal pluripotent stem cells, resembling archeocytes, within 24 h of initiating metamorphosis. By 36 h, some of these labeled archeocyte-like cells have differentiated into choanocytes that will form the first postlarval choanocyte chambers. Non-labeled cells also contribute to these primary choanocyte chambers, consistent with these chambers being a chimera of multiple transdifferentiated larval cell types and not the proliferation of a single choanocyte precursor. Moreover, cell proliferation assays demonstrate that, following the initial formation of choanocyte chambers, chambers grow at least partially by the proliferation of choanocytes within the chamber, although recruitment of individual cells into established chambers also appears to occur. EdU labeling of postlarvae and juveniles reveals that choanocyte chambers are the primary location of cell proliferation during metamorphosis.

**Conclusion:**

Our results show that multiple larval cell lineages typically contribute to formation of individual choanocyte chambers at metamorphosis, contrary to previous reports in other species that show sponge choanocyte chambers form clonally. Choanocytes in postlarval and juvenile *A. queenslandica* chambers can also divide, with choanocyte chambers being the primary location of cell proliferation. Interestingly, the level of cell proliferation varies greatly between chambers and appears to be contingent on the size, location and developmental state of the chamber. Small chambers on the periphery of the body tend to possess more dividing cells. As choanocytes can also dedifferentiate into archeocyte-like cells, cell proliferation in chambers may not only contribute to chamber growth and self-renewal but also increase the number of pluripotent archeocytes.

## Background

Sponges are sessile, aquatic animals that possess internal ciliated choanocyte chambers that pump seawater through an aquiferous network of canals, which connect with the external environment through a huge array of pores and enable the capture of food and the removal of waste [1, 2]. As sponges are recognized as one of the oldest extant metazoan phyla [3–5], their choanocytes have been viewed traditionally as a symplesiomorphic trait shared with choanoflagellates that has been lost in most other metazoans [6, 7]. Choanocytes and choanoflagellates are of strikingly similar size and morphology, with both being small and having an apical flagellum surrounded by a collar of microvilli [6–13]. Collared cells have also been found in a number of protozoans [14, 15] and some metazoans [16, 17], but these have been largely considered analogous due to their differences in morphology and function; some have proposed that this is also the case for choanoflagellate and sponge choanocytes [18].

Sponges have remarkable regenerative abilities and the capacity to continually remodel their body shape. These abilities appear to be largely achieved through their two major stem cell types, archeocytes and choanocytes [2, 19, 20]. Archeocytes are multifunctional, amebocytic cells that are pluripotent and capable of self-renewal; they are likely the predominant stem cell in most demosponges [21–25]. Choanocytes are also highly proliferative and can transdifferentiate into somatic and germ cells in a range of contexts including tissue regeneration, juvenile growth and remodeling, and sexual reproduction [1, 19, 23, 26–30]. Unlike many other metazoan stem cells, both archeocytes and choanocytes have additional specializations and physiological functions, including the capture of food by choanocytes and transportation of nutrients by archeocytes [1, 31]. Combining these observations with the structural similarity of sponge choanocytes with choanoflagellates, it has been proposed that this cell type may be the original stem cell in sponges and possibly metazoans [2, 23].

Given the importance of the choanocyte in the functioning, maintenance, regeneration and repair of the sponge body plan as well as their similarity with choanoflagellates, we sought to determine the ontogenetic origin of this cell type and the chambers they construct. In many demosponges, choanocyte chamber formation is thought to occur by differentiation of a single archeocyte into a choanocyte progenitor cell, which then proliferates to form a mature choanocyte chamber [31–33]. In demosponges, choanocyte chambers often form during metamorphosis, although there are exceptions where chambers are present in larvae (reviewed in [34]). Sponge metamorphosis in many cases involves the differentiation and transdifferentiation of larval cells into juvenile cells [28, 35–38]. However, the fate of larval cells during sponge metamorphosis appears to vary between species with descriptions ranging from most larval cells undergoing autolysis [39] and being phagocytosed by archeocytes [40, 41] to specific larval cell types giving rise to specific juvenile cell types (e.g., larval epithelial cells transdifferentiating into juvenile choanocytes [35, 36, 38]). In general, it is unclear how specific larval cell types and lineages contribute to the genesis of the juvenile body plans, including the formation of choanocyte chambers.

Here, we use cell lineage tracers, antibodies and cell proliferation markers to follow the ontogeny of choanocytes and choanocyte chambers during metamorphosis in *Amphimedon queenslandica.* Although it is known in this species that larval epithelial cells transdifferentiate into choanocytes and other cell types at metamorphosis [28, 36, 42], the specific steps and timings involved in the contribution of larval cells to choanocyte chamber development have not been determined. We show here that the first choanocyte chambers begin forming in *Amphimedon* at about 36 h after the initiation of metamorphosis. The number and size of these chambers continue to grow, and at around 72 h after the initiation of metamorphosis, a functional aquiferous system forms. Cell-tracing experiments reveal that choanocyte chambers often form by contributions from multiple larval cell lineages and proliferation of choanocyte progenitors. Continuous proliferation and late recruitment of individual choanocytes contribute to the further growth of these chambers. These results demonstrate that in *Amphimedon* and potentially other sponges, choanocyte chambers are not always clonal.

## Methods

### Sample collection

Adult *A. queenslandica* were collected and maintained in flow-through aquaria at the University of Queensland Heron Island Research Station (Great Barrier Reef Marine Park Authority permit G12/35053.1). Larval collection followed the protocol of [43] where adult sponges were induced to release larvae by mild heat treatment (1–2 °C above ambient temperature) for less than 2 h. These were collected into a beaker and left for 8 h to allow development of competency to settle and metamorphose [44].

Competent larvae were placed in 6-well plates with 10 ml of 0.2-μm filtered seawater (FSW) for 4 h in the dark with live coralline algae *Amphiroa fragilissima*, which strongly induces settlement and metamorphosis [45]. After 4 h, larvae settled on *A. fragilissima* were removed using fine forceps (e.g., Dumont #5) and resettled on to round coverslips placed in a well with 2 ml FSW in a 24-well plastic plate, with 3 postlarvae placed on each coverslip. These resettled postlarvae ball up and take the form similar to a newly settled larva. In terms of recording the time points of metamorphosis, we used this placement of newly settled postlarvae on the coverslips as the starting point of metamorphosis referred to as the 0 h postresettlement (hpr) stage, although they had originally settled on *A. fraglissima* up to 4 h before this time. Metamorphosis from a resettled larva to a functional juvenile takes approximately 72 hpr [28, 42].

### Immunohistochemistry

Postlarvae and juveniles on the coverslips were fixed according to [46]. Immunohistochemistry followed the protocol described in [28], using the antibodies against phospho-histone H3 [pSer10] (rabbit, 1:500, Abcam ab5176), acetylated-∂-tubulin (mouse 1:500, Sigma-Aldrich T6793) and tyrosinated-∂-tubulin (mouse 1:500, Sigma-Aldrich T9028). For secondary antibodies, we used AlexaFluor 488 (anti-rabbit or anti-mouse. 1:200, Molecular Probes), AlexaFluor 568 (anti-rabbit or anti-mouse. 1:200, Molecular Probes) and AlexaFluor 647 (anti-rabbit or anti-mouse, 1:200, Molecular Probes). AlexaFluor 488-conjugated phallacidin (1:25, Molecular Probes), which is generally used to label filamentous actin, was used as a counterstain to label F-actin-enriched cells in the inner cell mass and epithelial layer in larvae. For all samples, nuclei were labeled with the fluorescent dye 4′,6-diamidino-2-phenylindole (DAPI; 1:1000, Molecular Probes) for 30 min, washed in PBST for 5 min and mounted using ProLong Gold anti-fade reagent (Molecular Probes). All samples were observed using the Zeiss LSM 510 META confocal microscope, and image analysis was performed using the software ImageJ.

### Cell tracking using CM-DiI

The lipophilic cell tracker CM-DiI (Molecular Probes C7000) was used to label ciliated epithelial cells as described in [28]. Competent larvae were incubated in 10 μM CM-DiI in FSW for 16 h. After incubation, the larvae were washed in FSW several times and were induced to settle and initiate metamorphosis for 4 h and reared until fixation. These specimens were stained with DAPI, mounted in ProLong Gold anti-fade reagent and observed as described above.

### Visualizing proliferation using EdU

To visualize cell proliferation, the thymidine analogue EdU (Click-iT EdU AlexaFluor 488 cell proliferation kit, Molecular Probes C10337) was used as previously described [28]. Early postlarvae were incubated in FSW containing 200 μM of EdU for 6 h to label S-phase nuclei. They were then washed in FSW and immediately fixed as described above. Fluorescent labeling of incorporated EdU was conducted according to the manufacture’s recommendations prior to DAPI labeling and mounting on to slides with ProLong Gold anti-fade reagent.

## Results

### Changes in ciliation patterns during metamorphosis

**Fig. 1:**
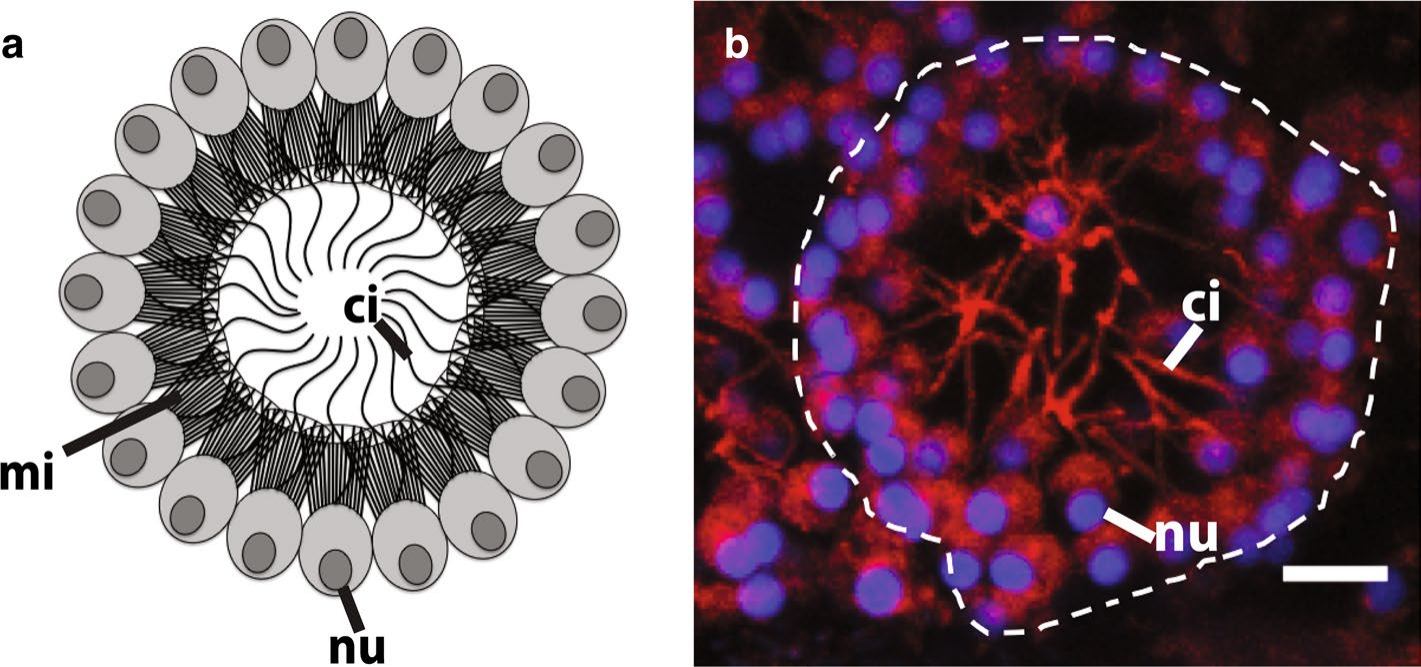
Anatomy of a choanocyte chamber in *A. queenslandica.* **a** A schematic drawing of a choanocyte chamber consisting of multiple choanocytes with a collar of microvilli and cilium pointing inward. **b** A confocal section of a choanocyte chamber in *A. queenslandica* consisting of multiple choanocytes with cilia pointing inward. Nuclei (nu) are stained with DAPI (*blue*); cilia (ci) are immunofluorescently labeled with an anti-acetylated alpha tubulin antibody as well as CM-DiI, which also label the cell body (*red*). *ci* cilium, *mi* microvilli, *nu* nucleus. *Scale bar* 5 μm

**Fig. 2:**
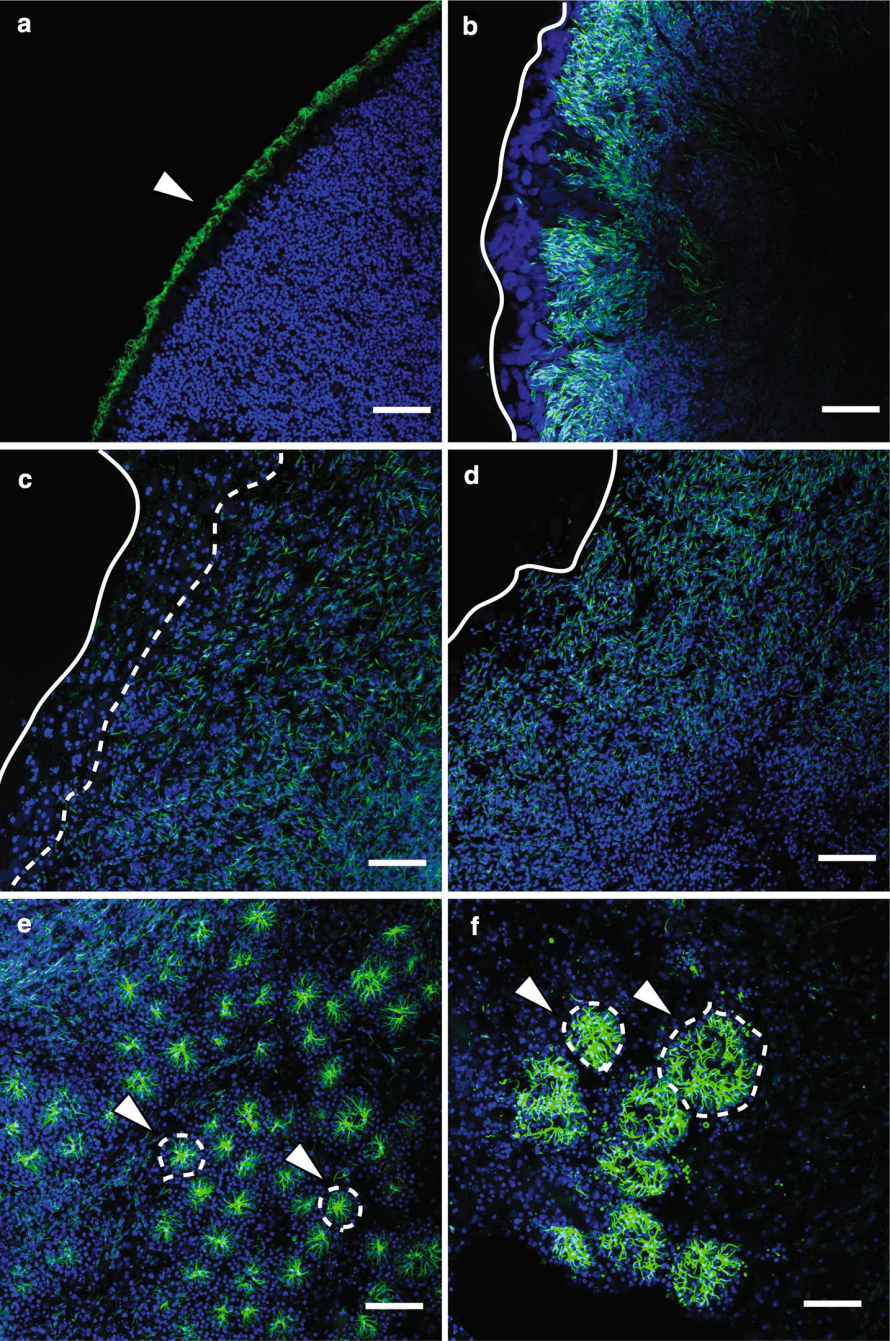
Ciliation pattern changes show choanocyte chamber formation timing during metamorphosis in *A. queenslandica.* Nuclei are stained with DAPI (*blue*), and cilia are immunofluorescently labeled with an anti-acetylated alpha tubulin antibody (*green*). **a** A confocal section of the external epithelium of a competent larva. Cilia occur predominantly on the apical surface of epithelial cells (*arrowhead*), while there is no evidence of ciliation in the inner cell mass. **b** 6 hour postresettlement (hpr). The epithelial integrity is lost [28]. Cilia are becoming resorbed into former epithelial cells [36], and thus, ciliation is no longer found on the external surface of the postlarva (white line). **c** 12 hpr. Overall ciliation is reduced. Cells containing the resorbed cilia are internalized (*white line*) and have not yet reached the edge of the body (*white line*). **d** 24 hpr. Cells containing resorbed cilia spread across the body (*white line*). **e** 48 hpr. Small choanocyte chambers are forming throughout the body (*arrowheads*, *circled by white dotted line*), with some cells containing resorbed cilia still visible. **f** 72 hpr. Larger choanocyte chambers are present (*arrowheads*, *circled by white dotted line*), with fewer cells containing resorbed cilia visible. *Scale bars* 25 μm

One of the distinct morphological features of choanocytes is the apical flagellum or cilium (Fig. 1). To visualize ciliated cells and to constrain the timing of choanocyte chamber formation during metamorphosis, fixed larvae and postlarvae were labeled with an anti-acetylated tubulin antibody, which recognizes ciliary microtubules, and DAPI, which binds to DNA and thus labels nuclei (Fig. 2). Ciliation occurs in columnar epithelial cells and flask cells in the competent larvae, consistent with previous observations using transmission electron microscopy (TEM) (Fig. 2a; [36]). In the early phase of metamorphosis, the cilia in most if not all cells appear to be partially resorbed, such that the length of the cilia appears much shorter than in the larvae (Fig. 2b), as previously observed by TEM [36]. These cells with resorbed cilia are enriched toward the center of the postlarva (Fig. 2c). By 24 hpr, cells containing resorbed cilia have spread out to the edge of the postlarval body (Figs. 2d). By 48 hpr, the first choanocyte chambers are visible, while there are still many cells containing resorbed cilia not associated with forming chambers (Fig. 2e). These newly formed choanocyte chambers are generally smaller and more uniform in size compared to juvenile and adult chambers. At 72 hpr, much larger chambers are detected, with adjacent chambers appearing to be interconnected (Fig. 2f). There are fewer individual cells with resorbed cilia present at 72 hpr compared to earlier stages.

### Multiple larval cell types including larval epithelial cells contribute to postlarval and juvenile choanocyte chambers

**Fig. 3:**
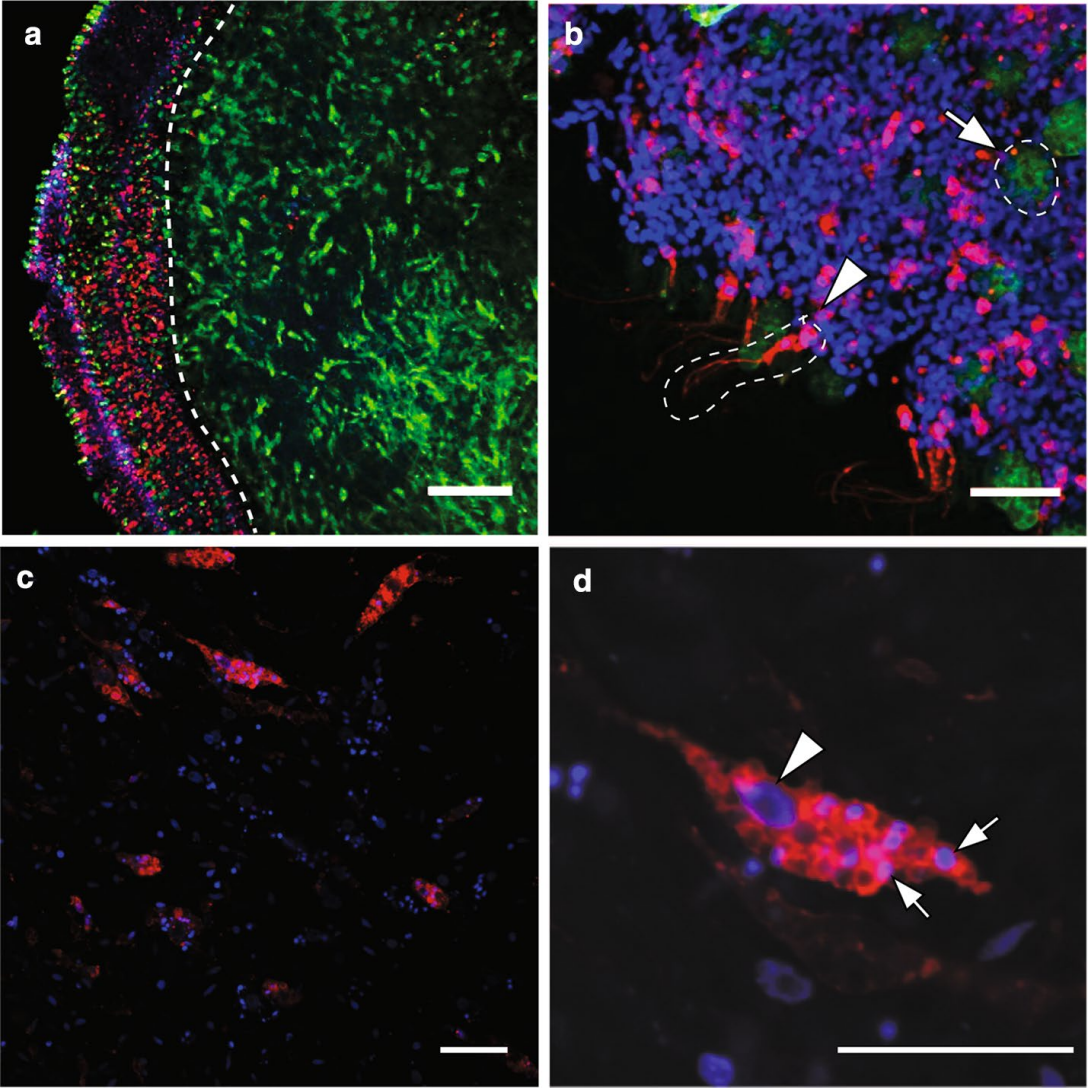
Larval epithelial cells transform into archeocytes during early metamorphosis. Nuclei are stained with DAPI (*blue*), and subsets of cells are labeled with CM-DiI (red) or phallacidin (*green*). **a** A confocal section of a competent larva labeled with CM-DiI. CM-DiI-labeled cells are present only in the epithelial layer of the larva. The *dotted line* indicates a boundary between the epithelial layer and subepithelial layer with the inner cell mass. **b** Confocal section of the larval epithelial layer showing ciliated columnar epithelial cells labeled with CM-DiI (*arrowhead*, *circled by dotted line*). Globular (spherulous) cells are non-specifically labeled by phallacidin (*arrow*, *circled by dotted line*). **c** Confocal section of archeocytes found during early metamorphosis in 24 hpr postlarvae. **d** A high-magnification image of an archeocyte. Small DAPI signals (*arrows*) are putative nuclear fragments obtained via phagocytosis of larval cells that underwent apoptosis at metamorphosis [28, 42]. The *arrowhead* shows the much larger and less heavily stained nucleus (with a nucleolus) of this archeocyte. *Scale bars* **a** 40 μm; **b**–**d** 15 μm

**Fig. 4:**
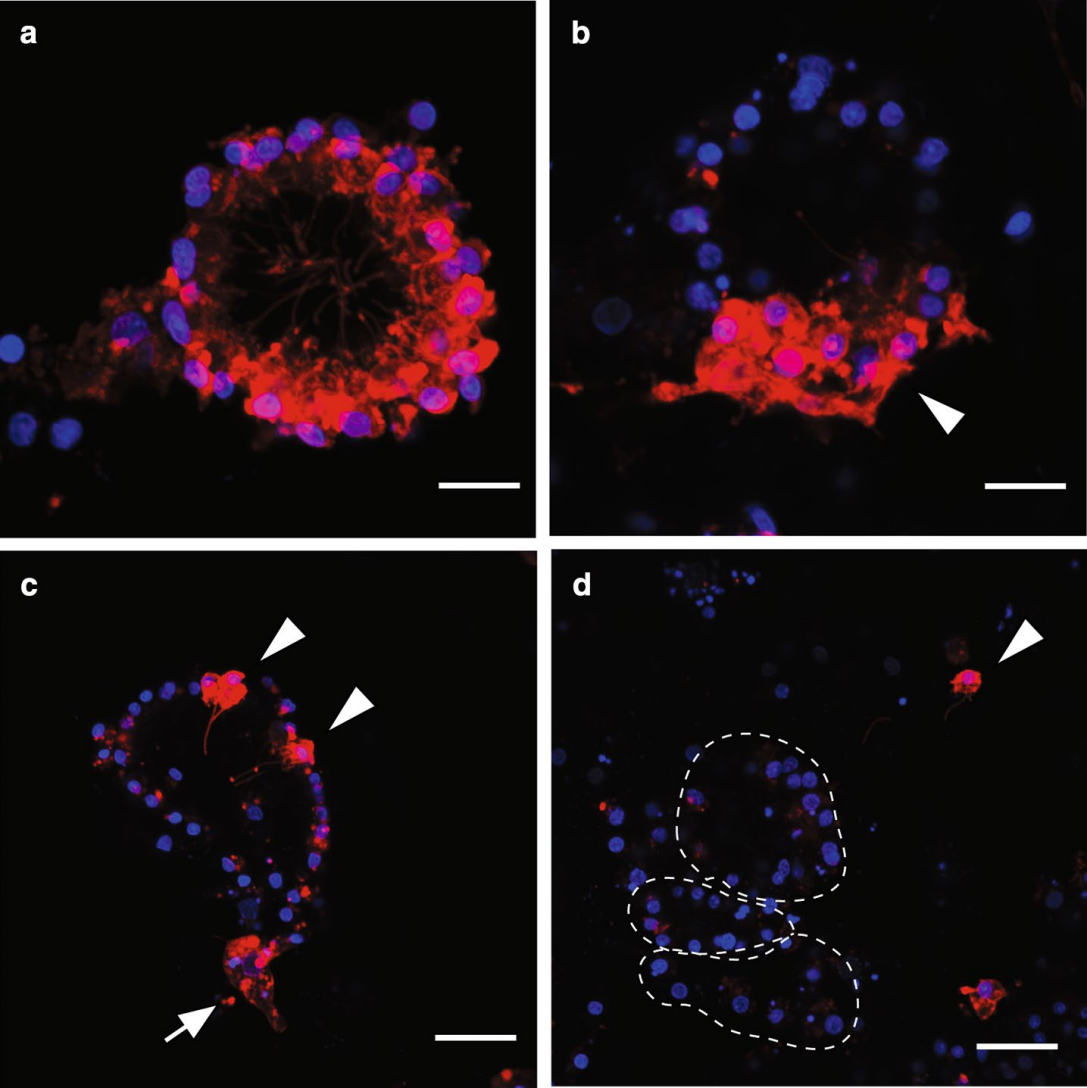
Evidence of non-clonal choanocyte chambers visualized by differential CM-DiI labeling of postlarval and juvenile choanocyte chambers. Nuclei are stained with DAPI (*blue*), and cells derived from the larval epithelium are labeled with CM-DiI (*red*). **a** A choanocyte chamber completely labeled with CM-DiI. This chamber could have formed by a single progenitor cell (clonal) or multiple CM-DiI-labeled progenitor cells (non-clonal). **b** A non-clonal choanocyte chamber indicated by a large cluster of CM-DiI-labeled (*arrowhead*) and unlabeled choanocytes in a single chamber. **c** Two small clusters of CM-DiI-labeled choanocytes (*arrowheads*) in a largely unlabeled chamber. The arrow shows a neighboring archeocyte also labeled with CM-DiI. (D) Unlabeled choanocyte chambers (*circled by dotted line*) and a single choanocyte in the extracellular matrix (*arrowhead*). CM-DiI-labeled vesicles are visible within the unlabeled choanocytes. *Scale bars* **a**, **b**, 5 μm; **c**, **d** 10 μm

As previous studies have shown that ciliated larval epithelial cells generate a range of juvenile cell types including choanocytes [28, 36], we sought to determine the specific contribution of larval epithelial cells to juvenile choanocyte chambers in *A. queenslandca*. Larval epithelial cells were labeled with CM-DiI and were traced through metamorphosis to investigate the clonality of postlarval choanocyte chambers. Only the epithelial layer has CM-DiI-labeled cells (Fig. 3a), with cilia clearly labeled by CM-DiI as well (Fig. 4b; [28]). CM-DiI does not label all larval epithelial cell types, including epithelial globular (spherulous) cells, which in Fig. 3b are labeled by phallacidin, but not CM-DiI. As such, the CM-DiI pulse-chase treatment used here follows only a subset of cells in the epithelial layer through metamorphosis, namely the ciliated columnar epithelial cells and the flask cells [28].

By 24 hpr, CM-DiI-labeled cells are no longer localized in the outer region of the postlarvae. The most abundant labeled cell type is an archeocyte-like cell (Fig. 3c, d), which has an ameboid shape and a large nucleolus, often possessing putative apoptotic nuclear fragments as observed in previous studies [28]. Indeed, by 12 hpr, there is no evidence of CM-DiI-labeled larval ciliated epithelial cells; thus, it appears that most ciliated epithelial cells had rapidly transformed into these archeocyte-like cells within a few hours of the initiation of metamorphosis. Only a subset of cells in early postlarvae are labeled with CM-DiI (Fig. 3c, d), and these all have an archeocyte-like morphology. Based on the size of nuclei and large nucleolus, many unlabeled cells also are likely to be archeocyte-like. Some of these presumably are derived directly from larval archeocytes and not a transdifferentiation event [28].

In late postlarvae (48–72 hpr), CM-DiI-labeled choanocyte chambers can be observed (Fig. 4). As no choanocyte-like cell types labeled with CM-DiI are detected in earlier stages of metamorphosis, these choanocytes appear to have differentiated from archeocyte-like cells prevalent in earlier stages of metamorphosis (Fig. 3c, d; [28]). Moreover, while there are some choanocyte chambers fully labeled with CM-DiI (Fig. 4a), only a portion of choanocytes is CM-DiI labeled in a majority of both newly formed small chambers (Fig. 4b) and larger more mature chambers (Fig. 4c). Although the origin of these unlabeled cells cannot be determined, this indicates that choanocytes in a given chamber originate from more than one larval cell lineage, resulting in a non-clonal choanocyte chamber. Partially labeled choanocyte chambers indicate two possible scenarios: (1) initial chamber formation involving choanocyte precursors of multiple origins, some of which were CM-DiI labeled, or (2) recruitment of CM-DiI-labeled choanocytes after initial chamber formation. The occasional presence of a single CM-DiI-labeled choanocyte not integrated into chambers (Fig. 4d) is consistent with the latter scenario, with these cells being future recruits into existing chambers. Completely unlabeled choanocyte chambers are also abundant in postlarvae and juveniles (Fig. 4d), consistent with non-labeled larval epithelial cells or non-epithelial larval lineages such as the larval archeocytes contributing to the postlarval/juvenile choanocyte population, as previously documented in [28].

### Clusters of choanocyte precursors appear by 30 hpr

**Fig. 5:**
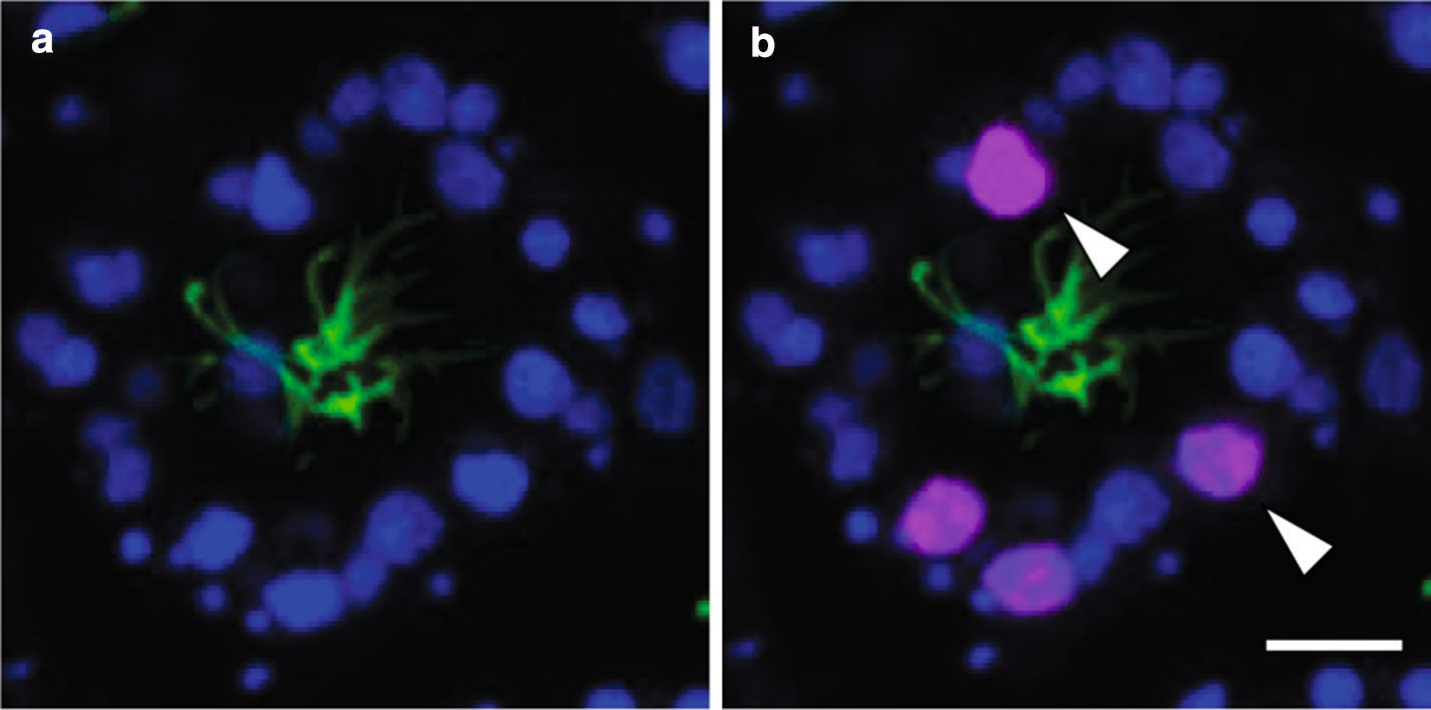
Choanocyte chambers labeled with anti-PH3 labeled nuclei in a 72-hpr juvenile. **a, b** Confocal sections of single choanocyte chambers. Nuclei are stained with DAPI (*blue*), and cilia are immunofluorescently labeled with an anti-acetylated alpha tubulin antibody (*green*), and mitotic nuclei are labeled with anti-phospho-histone H3 antibody (*magenta*). Note in **b** that a subset of choanocytes in the chamber is PH3 positive, indicative of mitosis (*arrowheads*). *Scale bar* 5 μm

**Fig. 6:**
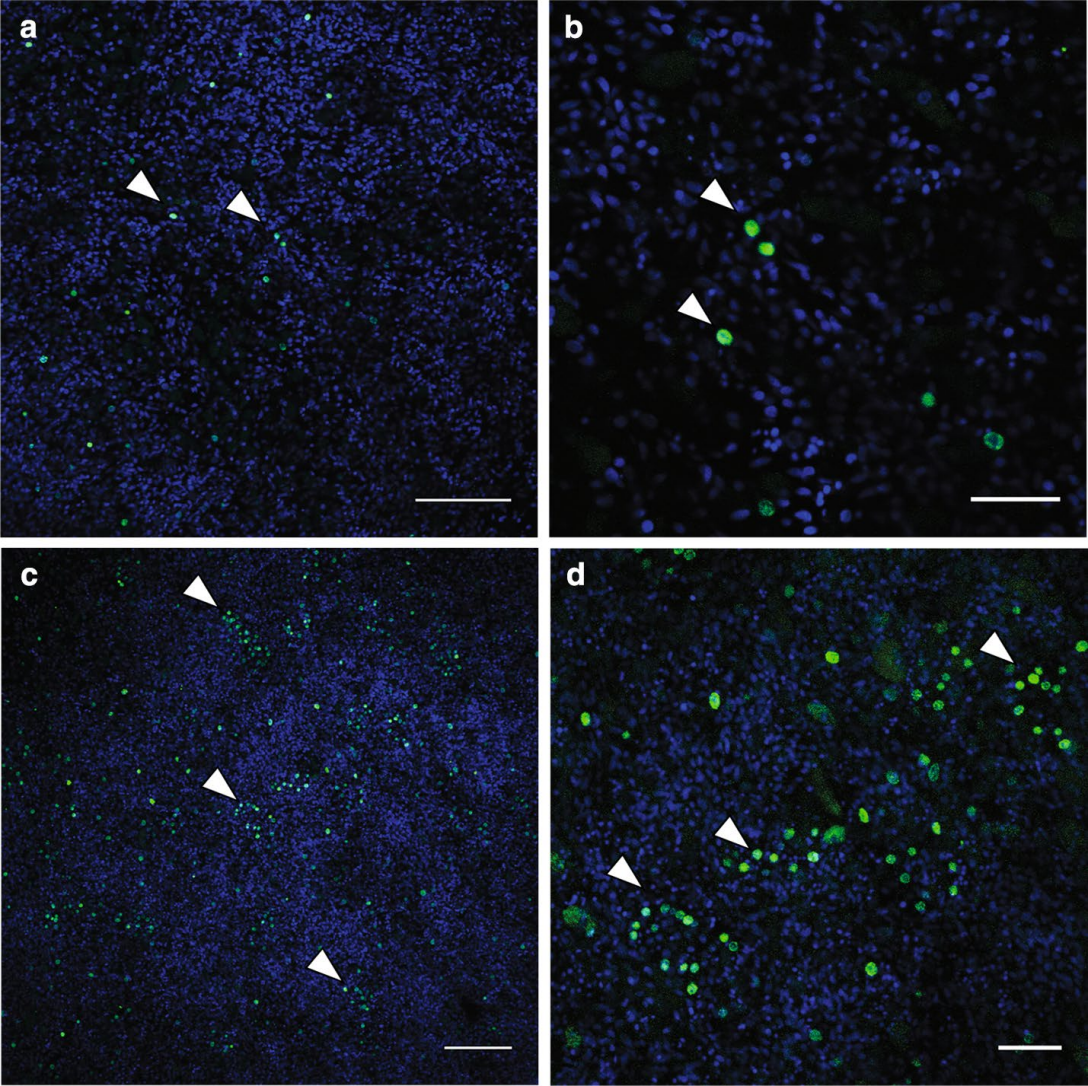
Proliferating nuclei labeled with EdU during early postlarval development. Nuclei are stained with DAPI (*blue*) and mitotic nuclei pulse labeled with EdU (*green*). **a**, **b** 24 hpr postlarva pulse labeled with EdU from 18 to 24 hpr. A small number of nuclei are labeled with EdU (*arrowheads*). **c**, **d** 30 hpr postlarva pulse labeled with EdU (from 24 to 30 hpr). An increase in proliferation rate can be observed, with clusters of EdU-positive nuclei evident as well (*arrowheads*). *Scale bars* **a**, **c** 40 μm; **b**, **d** 15 μm

**Fig. 7:**
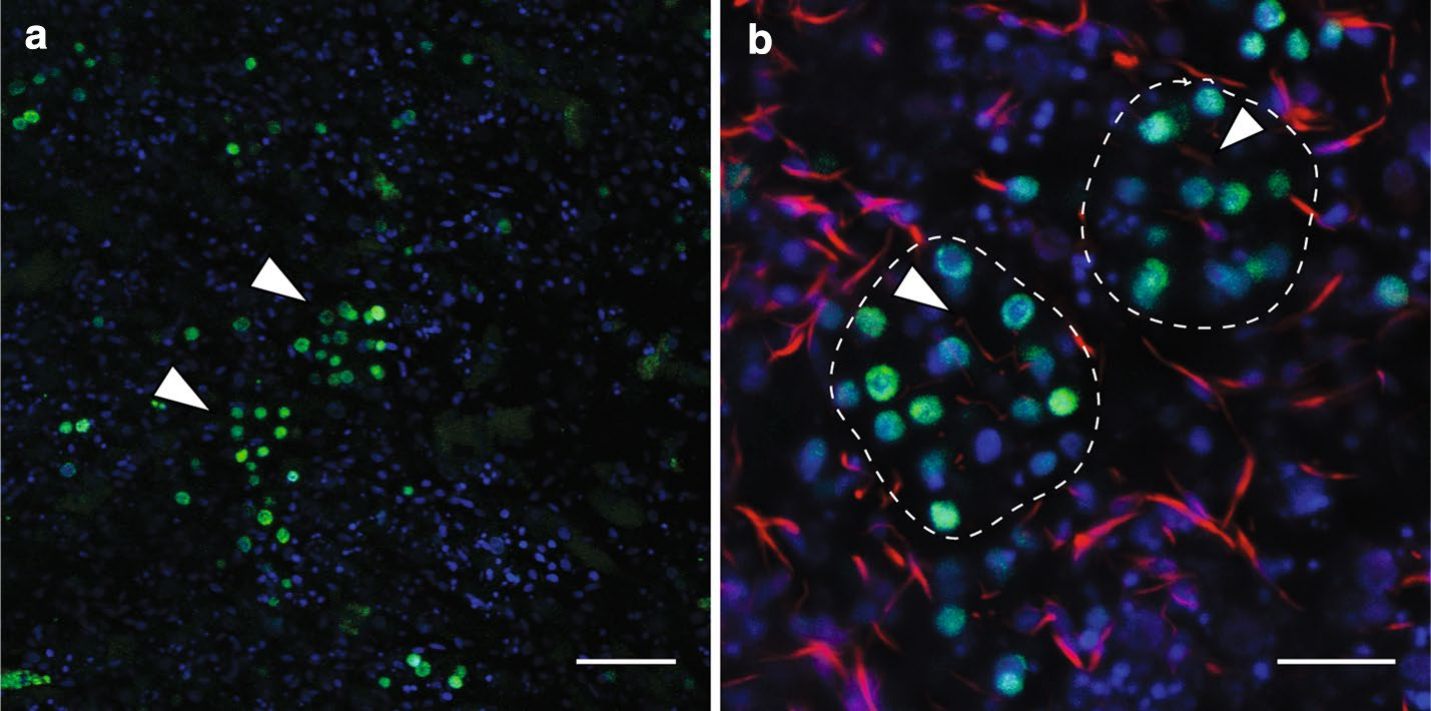
Cells in early choanocyte chambers labeled with EdU. Nuclei are stained with DAPI (*blue*) and EdU (*green*), and cilia are immunofluorescently labeled with an anti-acetylated alpha tubulin antibody (*red*). **a** A confocal section of a 36 hpr postlarva pulse labeled with EdU from 30 to 36 hpr. Clusters of EdU-positive nuclei are observed. **b** A high-magnification image of clusters of EdU-positive cells (putative early choanocyte chambers (*circled with dotted line*) in a 36 hpr postlarva). At this stage, the cilia (*arrowheads*) are observed inside early choanocyte chambers. *Scale bars* **a** 20 μm; **b** 10 μm

Cell division in choanocyte chambers first was visualized by using an anti-phospho-histone H3 (PH3) antibody that labels mitotic cells [47] and anti-acetylated tubulin antibody to label cilia of choanocytes (Fig. 5). To identify periods and regions of high cell proliferation during postlarval development, we then used sequential 6-h incubation windows of EdU during metamorphosis. A large proportion of cell proliferation detected by this method localized to regions where choanocyte chambers appear to be forming (Figs. 6, 7). In the first 24 h of metamorphosis (24 hpr), cell proliferation appears to be rare based on the number of nuclei labeled with EdU (Fig. 6a, b). Most of the EdU-labeled nuclei are large, consistent with archeocyte-like cells being the predominant proliferating cell type at this stage; there is no evidence of choanocytes or choanocyte chambers at 24 hpr. Between 24 and 30 hpr, there is a large increase in proliferating cells (Fig. 6c, d), with many EdU-labeled cells clustered together in a sphere-like pattern similar to a primary chamber. Although ciliation within these clusters was not observed at this stage, these labeled nuclei are markedly smaller than those observed in the archeocyte-like cells and more similar to choanocytes. Between 30 and 36 hpr, the proliferating cell clusters are forming a sphere (Fig. 7a) with some ciliation occurring inside these clusters (Fig. 7b). These localized clusters of EdU-labeled cells appear to be choanocyte chamber progenitors and suggest that the clusters of proliferative cells observed at 30 hpr are early-forming choanocyte chambers.

### Different rates of proliferation occur in juvenile choanocyte chambers

**Fig. 8:**
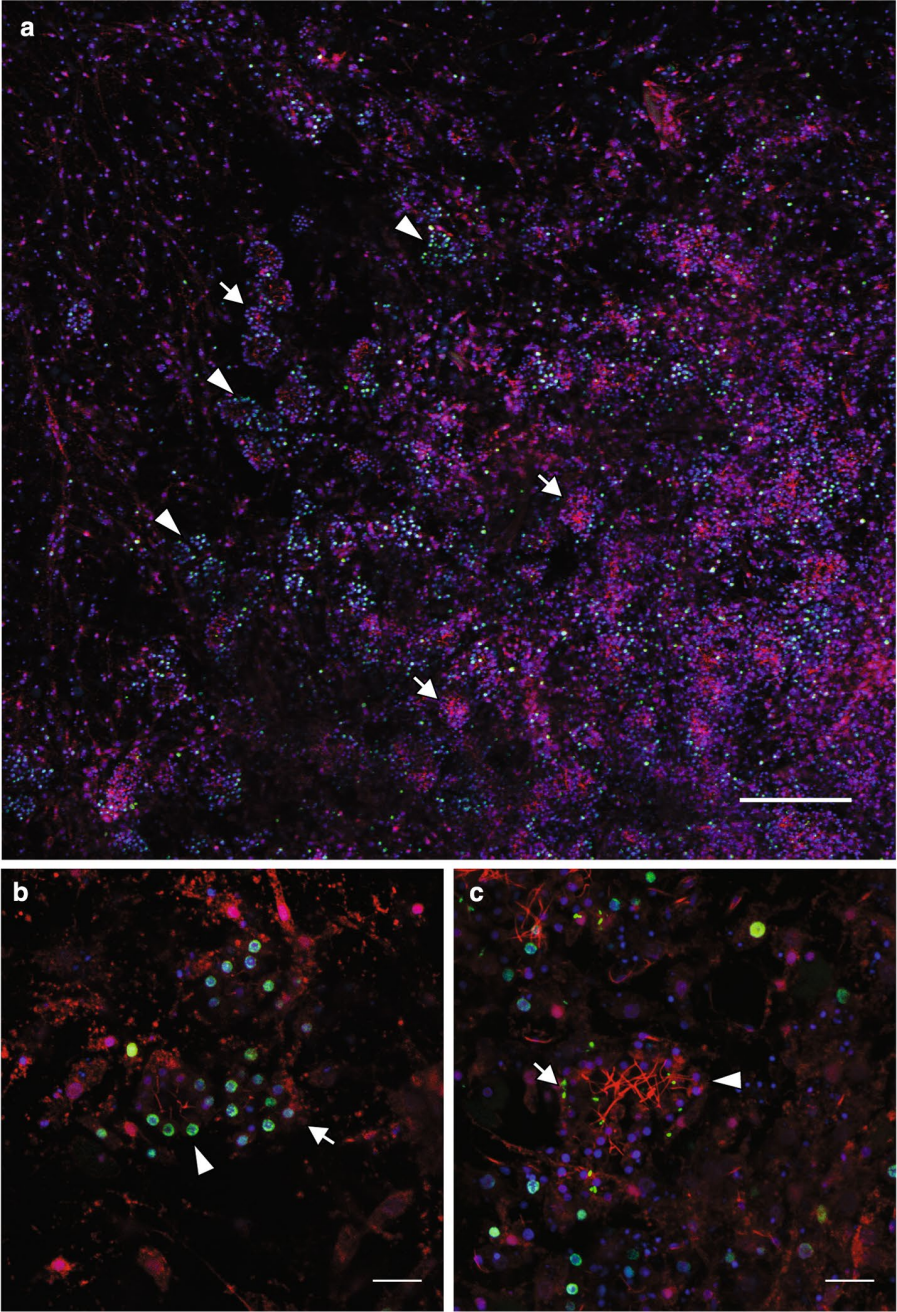
EdU labeling in juvenile choanocyte chambers shows variation in proliferation rates of individual chambers. Nuclei are stained with DAPI (*blue*) and EdU (*green*), and cilia and cytoplasm immunofluorescently labeled with both anti-acetylated alpha tubulin antibody and anti-tyrosinated alpha-tubulin antibody (*red*). **a** Juvenile labeled with EdU from 72 to 78 hpr. The right bottom corner of the micrograph is closer to the center of the juvenile, and the *top left corner* is the outer edge of the juvenile. Many EdU-positive nuclei are found throughout the juvenile body. There are variations in the number of EdU labeling found in chambers ranging from little to no EdU signals (*arrows*) to many or all nuclei labeled with EdU (*arrowheads*). **b** Choanocyte chambers with numerous EdU labeling. These appear to be early choanocyte chambers with relatively short cilia (*arrowhead*) or little to no cilia (*arrow*). **c** A mature choanocyte chamber with no EdU labeling (*arrowhead*). The small punctate EdU signals (*arrow*) are presumably proliferating bacteria. *Scale bars* **a** 80 μm; b, **c** 10 μm

Metamorphosis is complete at about 72 hpr when a functional aquiferous system is established [42]. At this stage, there are numerous choanocyte chambers of varying size and shape (Fig. 8). In general, most newly forming or early-stage choanocyte chambers have a large proportion of their cells labeled with EdU (Fig. 8b), but rarely are all the cells labeled. The smaller chambers with a larger proportion of EdU-labeled cells tend to be enriched toward the outer edge of the juvenile (Fig. 8a). Larger and presumably more mature choanocyte chambers generally have very little EdU-labeling and tend to be more centrally located (Fig. 8c), suggesting that variation in proliferation rates in these juvenile choanocyte chambers in *Amphimedon* may be dependent on size, location and developmental state.

## Discussion

In this study, we investigated the formation and growth of choanocyte chambers during metamorphosis of the demosponge *A. queenslandica*. This process begins with ciliated larval epithelial cells and probably other larval cell types transforming into pluripotent archeocyte-like cells within a few hours of the larva first settling and commencing metamorphosis. Archeocyte-like cells appear to be the prevalent cell type during the first 24 h of metamorphosis, suggesting most larval cells that are maintained during this transition transform into archeocytes [28]. Some of these cells then differentiate into a range of postlarval cell types, including choanocytes. By following the fate of CM-DiI-labeled larval epithelial cells, we demonstrate here that choanocyte chambers in juvenile *Amphimedon* can be comprised of cells from multiple larval origins. There were also chambers that were completely labeled with CM-DiI, indicating that these chambers were either generated clonally from a single progenitor cell or formed from multiple cells that were labeled with CM-DiI. The sources of non-labeled choanocytes include archeocytes from the larval inner cell mass [28].

### Multiple mechanisms underlie choanocyte chamber formation and growth

Despite the importance of choanocytes in many aspects of a functional sponge, including the stem cell system, the ontogeny of choanocyte chambers during metamorphosis has not been studied in great detail in demosponges. One of the best-described cases of choanocyte chamber development in demosponges is during asexual reproduction in *Ephydatia fluviatilis*. In this case, choanocyte chambers form clonally from the proliferation of a single archeocyte precursor [32, 48]. However, little is known about chamber formation during metamorphosis in *E. fluviatilis*. During this process in *A. queenslandica*, it appears that at least some primary chambers are not derived from a single progenitor cell; some chambers may be clonally derived. Recognizable choanocyte chambers first appear around 36 hpr. This corresponds to a period of a marked increase in cell proliferation, which is enriched in areas where chambers appear to be forming. Chamber growth continues through the formation of the functional juvenile aquiferous system, which occurs at about 72 hpr.

Cell division of choanocytes within juvenile *A. queenslandica* chambers is a contributing factor to the growth of the chamber, as observed in other sponges [31, 33]. In addition, in *A. queenslandica* there is also evidence that chamber growth occurs by recruitment of individual choanocytes. Individual CM-DiI-labeled choanocytes that are not associated with a chamber are observed in the *Amphimedon* postlarvae and juveniles (Fig. 4d). In addition, there are chambers comprised of a small number of CM-DiI-labeled choanocytes (2–3 cells) separated by multiple unlabeled cells (Fig. 4c). As observed in the EdU-labeling experiments, the cells involved in choanocyte chamber formation are highly proliferative, suggesting that these CM-DiI-labeled choanocytes are individual choanocytes that have been incorporated via recruitment into mature choanocyte chambers.

**Fig. 9:**
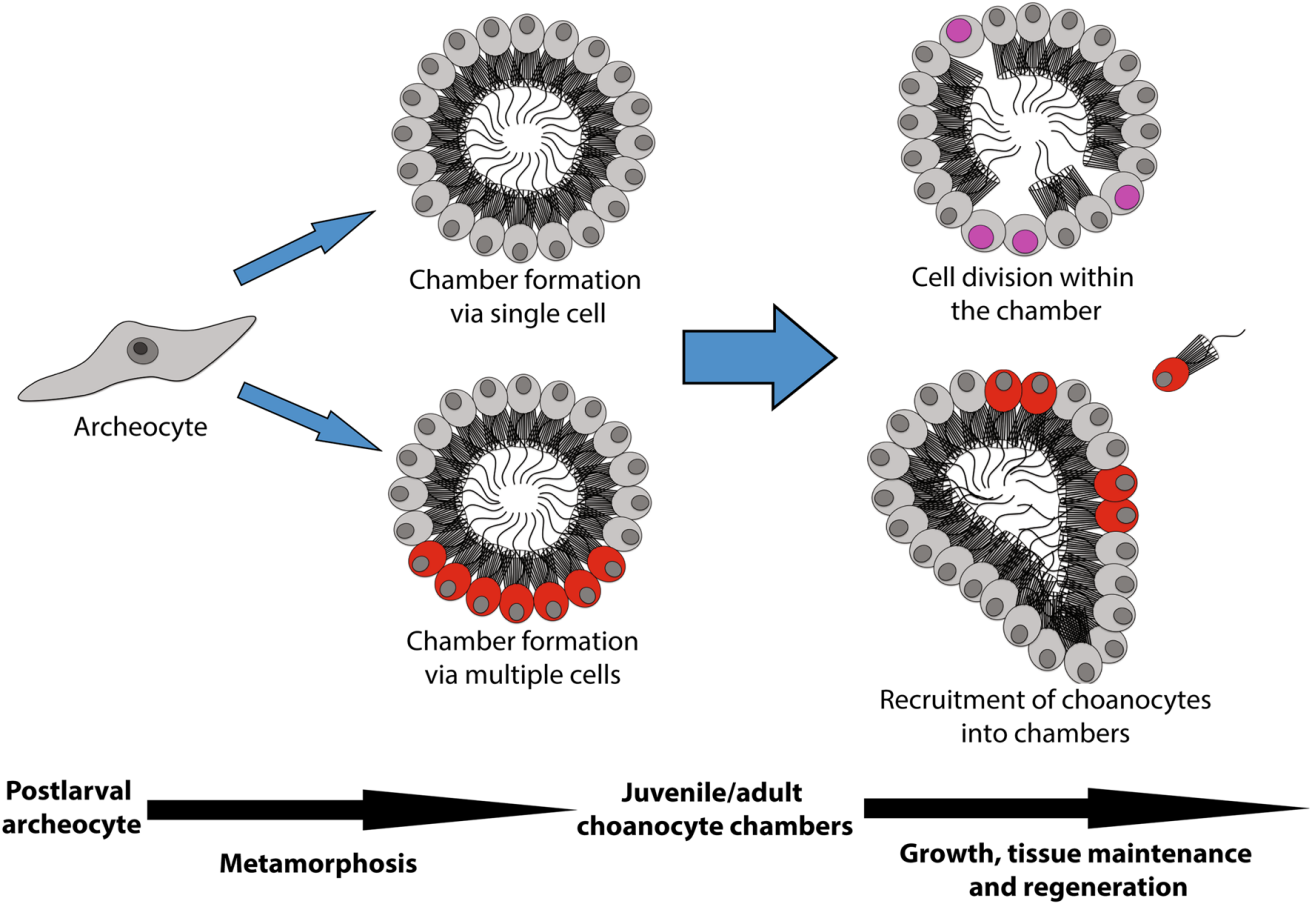
Proposed model of choanocyte chamber formation in *A. queenslandica.* During metamorphosis, postlarval archeocytes form choanocyte chambers from either a single progenitor cell or multiple cells. Choanocyte chambers grow by proliferation of choanocytes within the chamber and by recruitment of choanocytes into mature chambers

Based on the variable patterns of CM-DiI labeling of chambers, we infer that chambers develop by multiple mechanisms that include (1) initial chambers that are either clonal or cell lineage chimeras, (2) localized proliferation in new and established chambers and (3) late recruitment of individual choanocytes into established chambers. In the first and third cases, choanocytes are derived from progenitor cells, which themselves originate by the transdifferentiation of larval cells early in metamorphosis or from archeocytes in the larval inner cell mass [28]. Although we cannot determine when individual CM-DiI-labeled cells become incorporated into a chamber or precisely how many larval cell lineages are involved, together these observations are consistent with choanocyte chamber development and growth in *Amphimedon* postlarvae and juveniles involving multiple larval cell lineages and developmental cell mechanisms (Fig. 9).

### Choanocytes are an essential part of the *Amphimedon* stem cell system

The high level of proliferation in choanocyte chambers in juveniles is consistent with this being a stem cell niche in *A. queenslandica*, comprised of cells that can undergo self-renewal and dedifferentiate into pluripotent archeocytes [28]. These abilities, combined with the observation that choanocytes can transdifferentiate into spermatocytes in other sponges [1, 27], suggest sponge choanocytes are also pluripotent. This proposition is supported by the expression of stem cell markers in *E. fluviatilis* [20, 49]. In *A. queenslandica*, choanocytes in juvenile chambers dedifferentiate into archeocytes that can give rise to a diversity of somatic cell types [28]. Currently, it is unclear whether choanocytes in all choanocyte chambers are dedifferentiating at equivalent rates or whether the level of cell proliferation in a given chamber determines a chamber’s ability to generate archeocytes.

Archeocytes migrate throughout the sponge body and differentiate into various cell types. Although these cells have the capacity for self-renewal, it is unclear whether this is sufficient to maintain a functional population as a sponge grows or regenerates [2]. In *A. queenslandica* postlarvae and young juveniles, choanocytes in chambers can be highly proliferative and are capable of dedifferentiating into archeocytes (this study; [28]), consistent with choanocyte chambers contributing to the replenishment of the archeocyte pool. This process is likely to be occurring in other sponges [29, 50]. Although the precise relationship between choanocyte proliferation and dedifferentiation is currently unknown, our study shows that individual chambers vary markedly in their state of proliferation, with some chambers undergoing extensive cell division, while others are quiescent. Since sponges have highly variable regenerative capacities, which can influence growth form characteristics (e.g., branching, encrusting and cryptic) and susceptibility to disturbance [51], the specific processes involved in this stem cell system may be variable between species [19, 26]. The stem cell system in *A. queenslandica* appears to be an inherently dynamic system that relies on context-specific regulation of proliferation and transdifferentiation of choanocytes and archeocytes.

## Conclusions

This study reveals that multiple larval cell lineages contribute to the initial formation of individual choanocyte chambers at metamorphosis in *A. queenslandica*. This is in contrast to other demosponges, where individual choanocyte chambers appear to be clonally derived from a single progenitor cell. Proliferation of choanocytes in the chamber and recruitment of individual choanocytes from outside the chamber appear to be the primary means by which newly established chambers grow in *A. queenslandica*, with choanocytes commencing mitosis about 30 h after the initiation of metamorphosis. The choanocyte is perhaps the most proliferative cell type in postlarvae and juveniles, although the level of cell proliferation varies greatly between chambers and appears to be contingent on the size, location and developmental state of the chamber. As choanocytes can also dedifferentiate into archeocyte-like cells, cell proliferation in chambers may not only contribute to chamber growth and self-renewal but also the replenishment of pluripotent archeocytes.

## Abbreviations

*A. fragilissima*: *Amphiroa fragilissima*
*A. queenslandica*: *Amphimedon queenslandica*
ci: cilium
mi: microvilli
CM-DiI: cell tracker CM-DiI
DAPI: 4′,6-diamino-2-fenilindol
EdU: click-iT EdU (5-ethynyl-2′-deoxyuridine) AlexaFluor 488 cell proliferation kit
*E. fluviatilis*: *Ephydatia fluviatilis*
FSW: filtered sea water
h: hours
hpr: hours postresettlement
nu: nucleus
PH3: phospho-histone H3 [pSer10]
TEM: transmission electron microscopy
